# REEDD-CR: Residential electricity end-use demand dataset from Costa Rican households

**DOI:** 10.1016/j.dib.2022.108829

**Published:** 2022-12-16

**Authors:** Jam Angulo-Paniagua, Luis Victor-Gallardo, Ignacio Alfaro-Corrales, Jairo Quirós-Tortós

**Affiliations:** School of Electrical Engineering, University of Costa Rica, San José, Costa Rica

**Keywords:** Electricity demand, End-use, Monitoring, Residential

## Abstract

End-use demand data availability is a catalyst for improving energy efficiency measures and upgrading electricity demand studies. Nevertheless, residential end-use public datasets are limited, and end-use monitoring is costly. The lack of electricity end-use data is even more profound in Latin America, where there are no public end-use datasets as far as the authors are concerned. Hence, we present the Residential Electricity End-use Demand Dataset of Costa Rica (REEDD-CR), containing the results of monitoring 51 Costa Rican households. The data set includes the aggregated and branch circuit measurements for every home with a sample time of 1 min for at least an entire week. The measurements were distributed all around the country. In addition, based on these sub-measurements, REEDD-CR includes a dataset of 197 load signatures composed of seven consumption and demand features for eight high-consuming appliances: refrigerator, stove, dryer, lighting, water heating, air conditioning, microwave, and washing machine. The features included on each load signature are average power, peak power, average daily events, average daily energy, day-use factor, night-use factor, and time of use. The single-appliance measurements used to calculate these load signatures are also part of the dataset. The release of REEDD-CR can serve as a tool for appliance modeling, demand disaggregation testing, feedback for energy demand models, and the overall upgrade of electricity supply and demand simulation studies with realistic and disaggregated data.

**Specifications Table**

**Table:** 

Subject	Energy
Specific subject area	Residential electricity end-use demand
Type of data	Table Dataset
How the data were acquired	Electricity demand monitoring at each household's main circuit panel.
Data format	Raw Analyzed Filtered
Description of data collection	Data were collected from each household's main circuit panel. The principal and branch circuits used a monitoring device to measure their electricity demand each minute for at least an entire week. After installing the equipment, the circuits were identified and labeled with the main end-uses they supply. The households are distributed across the country. Their selection depended on their owners' or renters' availability and willingness to participate in the project. The measurements that monitored a single high-consuming end-use were used to calculate seven energy and power demand features for their corresponding appliance.
Data source location	Country: Costa Rica
Data accessibility	Repository name: REEDD-CR: Residential Electricity End-use Demand Dataset from Costa Rican households Data identification number: DOI:10.17632/6zrscrf3yv.1 Direct URL to data: https://data.mendeley.com/datasets/6zrscrf3yv
Related research article	*-*

## Value of the Data

• The data can be used for appliance modeling, testing of demand disaggregation algorithms, electricity demand estimations, and the overall upgrade of electricity demand simulation studies.
• Helpful for researchers, utilities, and policymakers working toward understanding consumers' behavior and end-use electricity consumption.
• Useful to understand the contribution of specific devices to the overall electricity consumption of a household.
• The dataset contributes to the limited number of public datasets with real end-use electricity measurements.
• Although measurements correspond to Costa Rican households, the dataset can be a reference for understanding appliance electricity consumption in other countries and regions as well.

## 1 Data Description

This paper presents the Residential Electricity End-use Demand Dataset of Costa Rica (REEDD-CR) dataset. It contains end-use electricity demand measurements of 51 Costa Rican households with a 1-minute sample time. Monitors were installed in each home to record the electricity demanded by the principal (i.e., aggregated) and branch (i.e., disaggregated or individual) circuits. Every measurement includes at least an entire week. Therefore, it consists of both weekdays and weekends. They are labeled with the corresponding appliances they represent.

In addition, REEDD-CR includes a set of load signatures (i.e., representation of an appliance's operation). Every device has a characteristic load signature. The load signature results from the appliance's working pattern, the conditions in which the devices operate (e.g., temperature and pressure), and the way users manipulate the devices (e.g., time of use and power levels) [1]. A method to represent an appliance's load signature is feature extraction [2]. This dataset has load signatures with seven features for eight common end-uses: refrigerator, stove, dryer, lighting, water heating, air conditioning, microwave, and washing machine. The features are related to the end-uses' power demand and energy consumption (i.e., average power, peak power, average daily events, average daily energy, day-use factor, night-use factor, and time of use).

REEDD-CR has three main groups of data, separated into different directories: *i)* sub-measurements, which correspond to all of the aggregated and disaggregated electricity demand measurements, *ii)* single appliance measurements, which has the end-use time series used to calculate the load signatures, and *iii)* the load signatures, that corresponds to the features calculated for each end-use. The following sections explain the three of them.

### 1.1 Aggregated and branch circuit measurements

These data correspond to the complete electricity demand measurements, including each home's principal and branch circuits. The dataset has a different file for each household in the *0_submeasurement* folder. The files' names have the form of *S_<n>.csv*. Here, *_<n>* corresponds to the household number. The first column corresponds to the measurement date and time in each file, and the remaining columns have the data monitored by each channel of the monitoring device. The first row enumerates the measurement channels, and the second row indicates the type of voltage supply that each circuit had. The voltage supply is coded as follows: *120* indicates that the circuit is connected to 120 V, *240* indicates that the circuit is connected to 240 V, and 120/240 is used for those circuits with a 240 V connection, but both phases were measured.

A separate folder named *0_labels* contains the labels for each sub-measurement (i.e., a list of the end-uses plugged into the corresponding circuit). The names of these documents have the form of *L_<n>.csv*, where *_<n>* corresponds to the number of the household. The measurements and labels with the same number denote the same household. The device used to collect the data in REEDD-CR has 14 measurement channels (see Section 2.1). In most cases, empty columns in the label files denote unused channels. These channels (or columns) have *null* or only zero values in the sub-measurement files. In a limited number of cases, it was not possible to identify the outputs for some branch circuits. These circuits were monitored but did not record any significant power consumption. This explains the noise (i.e., very low values) in some measurements with no labeling.

**Fig. 1:**
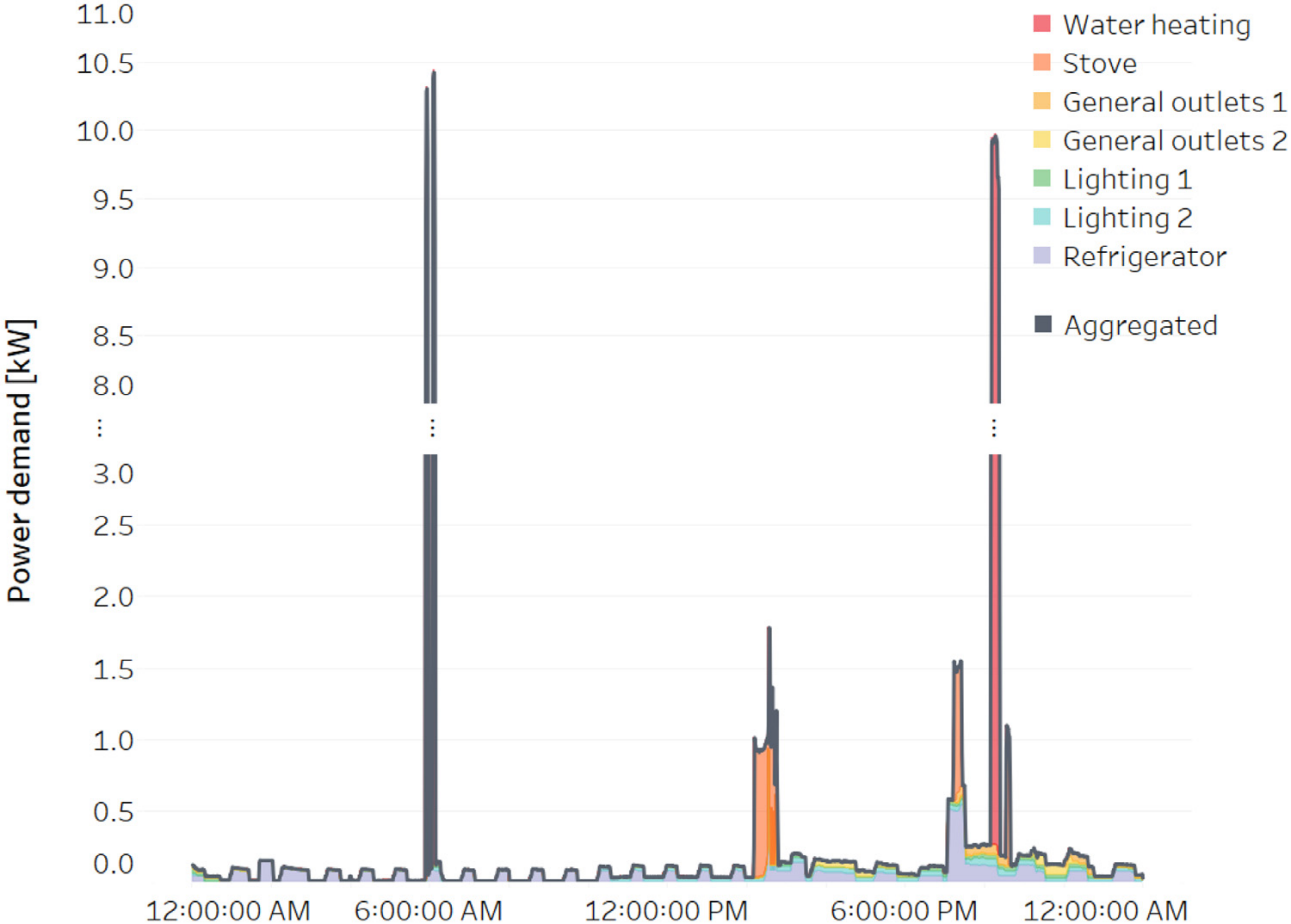
Aggregated and sub-metered power demand on household number one of REEDD-CR on October 25^th^, 2018.

To exemplify the data available in REEDD-CR, Fig. 1 shows the power demand measurements of a typical day in household number 1 of the dataset.

### 1.2 Single end-use measurements

These data have measurements corresponding to individual end-uses. The time series were selected from those sub-measurements with only one end-use or one dominating end-use. In addition, the files in this folder (named *1_single_appliance_measurements*) are the raw data used to calculate the load signatures. There is a folder for each end-use: air-conditioning (*AC*, 1 measurement), dryer (*D*, 12 measurements), lighting (*L*, 62 measurements), microwave (*M*, 20 measurements), refrigerator (*R*, 16 measurements), stove (*S*, 32 measurements), washing machine (*WM*, 7 measurements), water heating (*WH*, 47 measurements). The names of the files have the following form: *<End-use>_<m>*. Here, *<End-use>* corresponds to the initial letter(s) of the end-use, and *<m>* enumerates the end-use sub-measurements.

**Fig. 2:**
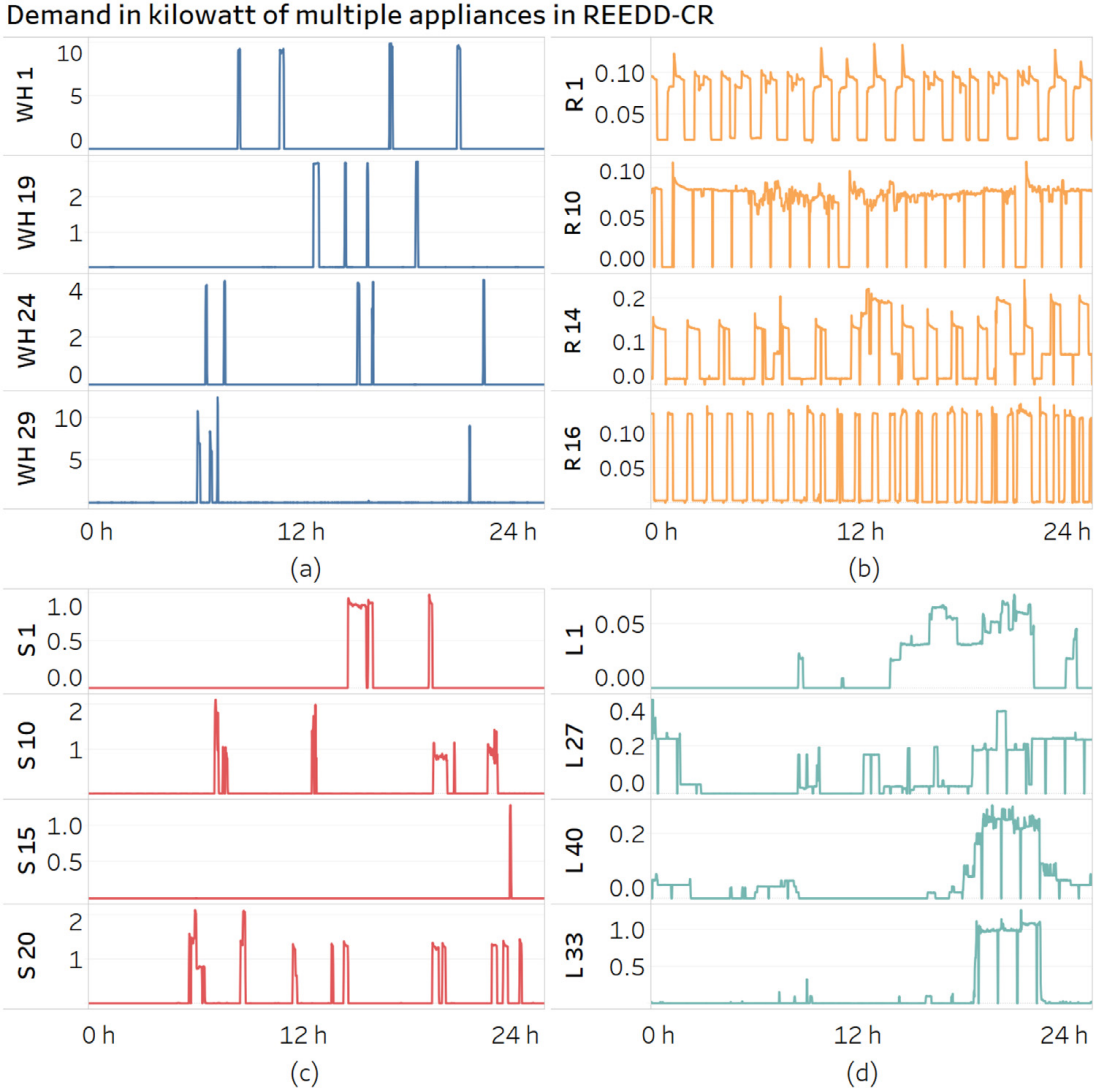
Sample of single end-use measurements from REEDD-CR of (a) Water heating – WH, (b) Refrigerator – R, (c) Stoves – S, and (d) Lighting – L.

Fig. 2 illustrates the type of single-appliance measurements available in REEDD-CR. Fig. 2a shows the power consumption of water heating systems, which occurs in short periods. In contrast, the refrigerators have a use pattern throughout the day (Fig. 2b). Stove (Fig. 2c) and Lighting (Fig. 2d) reflect the uses according to the time of the day, i.e., when it gets dark and meal preparation.

### 1.3 Load signatures (2_load_signatures)

The last group of data in REEDD-CR contains the load signatures that are part of the dataset. Our definition for each feature in the load signature was:

• Average power (*AveragePower_W*): this feature represents the average power in periods in which the device is active (i.e., ON state). This feature in the dataset is given in watts. For every feature, a device was considered to be ON whenever its power demand was above 3 W.
• Peak power (*Peak_power_W*): this value corresponds to the highest value of power recorded in measurements for every specific end-use. This feature in the dataset is given in watts.
• Daily number of events (*Average_daily_events*): this characteristic quantifies the average number of times each device is activated (i.e., changes from OFF to ON) during a day.
• Daily energy consumption (*Average_daily_energy_Wh*): this feature represents the average energy consumption of each device during a day of operation. This feature in the dataset is given in watt-hour.
• Use factor (*Use_factor*): this variable quantifies the proportion of the day in which the device is active (i.e., ON) during the day. The dataset separates this feature into the day use factor (*Day_use_factor*, from 6:00 am to 6:00 pm) and the night use factor (*Night_use_factor*, from 6:00 pm to 6:00 am). The daily use factor is expressed as a number between 0 and 1, and the day and night factors are a number between 0 and 0.5.
• Time of use (*Time_of_use_min*): it corresponds to the average time a device is used whenever it is active (i.e., the time elapsed between a change from OFF to ON and later from ON to OFF). This feature is given in minutes.

Table 1 presents the average values and standard deviation for each feature and device included in REEDD-CR's load signatures.

**Table 1:** Average values and standard deviation for the load signatures of every appliance considered in REEDD-CR.

	Device	Average power [W]	Peak power [W]	Average daily events	Average daily energy [Wh]	Day use factor	Night use factor	Time of use [min]
**Average values**	Refrigerator	129.66	265.40	28.05	1626.61	0.27	0.26	27.92
	Stove	839.14	3035.90	6.33	1432.05	0.06	0.04	23.14
	Dryer	2362.94	4606.00	1.26	1333.11	0.02	0.003	29.26
	Lighting	83.14	529.05	14.48	1012.58	0.25	0.31	103.93
	Water heating	3753.86	6491.49	6.37	2056.38	0.02	0.01	19.62
	Air conditioning	866.63	1391.50	1.17	4781.33	0.13	0.08	259.29
	Microwave	1278.20	1771.19	5.18	234.88	0.004	0.002	2.19
	Washing machine	652.86	911.971	2.62	164.89	0.01	0.003	4.95
**Standard deviation**	Refrigerator	16.41%	17.63%	24.96%	47.30%	36.02%	39.51%	42.49%
	Stove	34.59%	37.56%	93.29%	98.15%	149.96%	236.46%	98.59%
	Dryer	26.77%	26.35%	67.55%	137.07%	117.96%	158.75%	103.07%
	Lighting	99.57%	117.18%	77.50%	77.81%	63.25%	41.90%	178.54%
	Water heating	40.88%	52.08%	91.75%	75.88%	59.21%	139.56%	342.92%
	Air conditioning	-	-	-	-	-	-	-
	Microwave	12.54%	20.07%	65.64%	78.70%	82.03%	66.33%	41.25%
	Washing machine	21.18%	20.29%	99.97%	105.08%	89.82%	135.15%	89.38%

## 2 Experimental Design, Materials, and Methods

Before proceeding with the measurements involved in REEDD-CR, several characteristics were pre-defined as desired in the dataset:

• To monitor the aggregated profile of every household.
• To include as many end-uses as possible in the monitoring, prioritizing the highest consuming appliances.
• To procure clock-synchronization among all the end-use sub-measurements and between them and the aggregate profile.
• To include measurements of both weekends and weekdays for every household.
• To contemplate a wide variety of households, including rural and urban locations, spread across the country.

The following sections describe the methods for obtaining REEDD-CR's sub-measurements and load signatures.

### 2.1 Sub-measurements

**Fig. 3:**
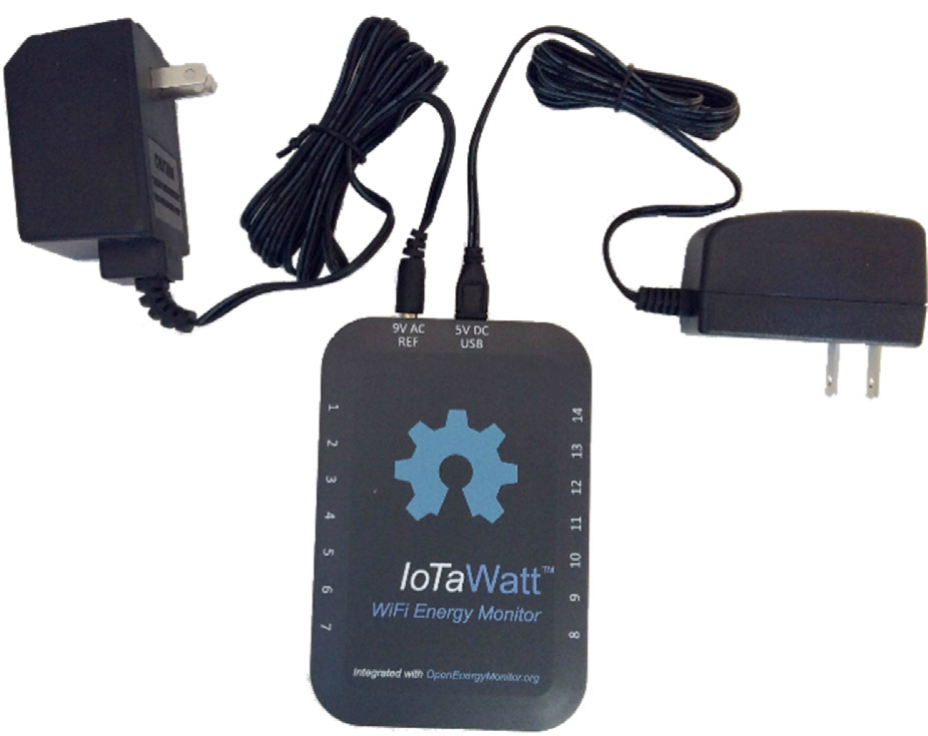
IoTaWatt, monitoring equipment used for REEDD-CR [3].

The monitoring device used for REEDD-CR was the IoTaWatt. This open monitor records electricity demand with 14 inputs connected to current transformers (CTs) that clip around each one of the wires of the circuits of interest [3]. In this case, the IoTaWatt was installed in the main circuit panel of each household. Therefore, the aggregated and branch circuits could be monitored with a single IoTaWatt. Fig. 3 shows the device.

**Fig. 4:**
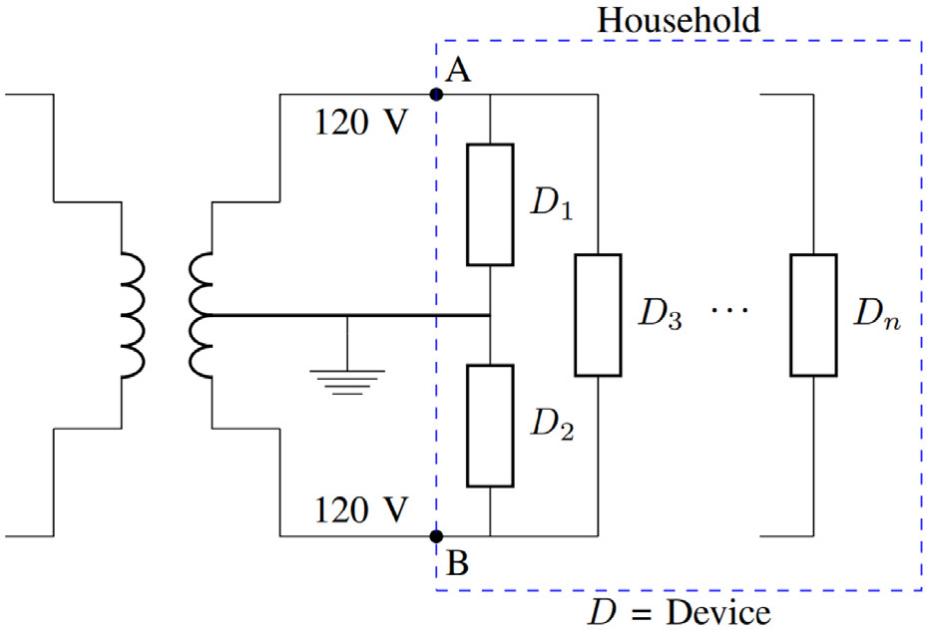
Regular residential configuration in Costa Rica.

Fig. 4 presents a typical configuration for residential electricity supply in Costa Rica. In the installation of REEDD-CR, two CTs were clipped around both main circuits for monitoring the aggregated power demand (node A and B in Fig. 4). The remaining CT inputs (12 total) monitored the branch circuits, corresponding to the end-uses of interest. Clock synchronization was a crucial advantage of monitoring from the main circuit panel with a single device. By using a single monitoring device, all measurements were collected simultaneously, avoiding complicated synchronization procedures.

Nevertheless, sub-metering faces several challenges. For instance, a singular branch circuit can supply multiple end-uses. In these cases, branch circuit identification is crucial. Therefore, labeling each circuit with its corresponding appliances and CT input was the first step during every installation.

Additionally, the available inputs were sometimes insufficient for the number of branch circuits (i.e., households with more than 12 branch circuits, in this case). The following considerations were made to solve this problem:

• Branch circuits with usually the highest energy consumption were prioritized (i.e., refrigeration, cooking, lighting, and water heating in the case of Costa Rica).
• When possible, two or more branch circuits corresponding to the same end-use (mainly lighting) were monitored with a single CT, i.e., one CT was clipped around more than one insulated wire.
• In most cases, for devices connected to 240 V, only one CT was used. Nevertheless, checking their specific supply system before proceeding with the installation was critical. For instance, there are stoves and oven combos with a 240 V connection. However, the stove section is connected to 120 V and the oven connected to 120 V. In these cases, two CTs are needed. Both situations were clarified in the circuit labeling during the monitoring process.

The monitoring process lasted for at least an entire week in each home, so weekdays and weekends were included—however, the starting day of the week varied. Urban and rural homes were part of the study. Both considerations were made aiming at diversifying the data set. Each measurement has a 1-minute sample time.

### 2.2 Load signatures

The load signatures for eight different appliances are represented by extracting seven relevant features from the sub-measurements. The feature selection incorporates characteristics related to the devices' power demand and time of use. This was done to have a comprehensive load signature of each device and differentiate those devices with similar power demands. The load signatures were calculated in those sub-measurements that monitored a single end-use or where the end-use of interest was easy to identify and extract (i.e., sub-measurements with multiple end-uses were discarded in this calculation).

## CRediT Author Statement

**Jam Angulo-Paniagua:** Conceptualization, Methodology, Data curation, Writing- Original draft preparation**. Luis Victor-Gallardo:** Conceptualization, Methodology, Data curation, Writing- Reviewing and Editing**. Ignacio Alfaro-Corrales:** Data curation. **Jairo Quirós-Tortós:** Conceptualization, Supervision, Methodology, Writing- Reviewing and Editing.

## Declaration of Competing Interest

The authors declare that they have no known competing financial interests or personal relationships that could have appeared to influence the work reported in this paper.

## Data Availability

REEDD-CR: Residential Electricity End-use Demand Dataset from Costa Rican households (Original data) (Mendeley Data).
